# Supplementary material for: Role and mechanism of NCAPD3 in promoting malignant behaviors in gastric cancer
Source: Front Pharmacol. 2024 Apr 22;15:1341039. doi: 10.3389/fphar.2024.1341039 (PMC11070777; doi:10.3389/fphar.2024.1341039)
Supplement: Supplementary file 11 [file DataSheet2.ZIP › GSEA/immunologic/my_analysis.Gsea.1599462003817/GSE27786_ERYTHROBLAST_VS_NEUTROPHIL_DN.html]

Details for gene set GSE27786\_ERYTHROBLAST\_VS\_NEUTROPHIL\_DN[GSEA]

|  || Dataset | filtered\_dataset.sample\_info.cls#WT\_versus\_NCAPD3\_MUT |
| Phenotype | sample\_info.cls#WT\_versus\_NCAPD3\_MUT |
| Upregulated in class | WT |
| GeneSet | GSE27786\_ERYTHROBLAST\_VS\_NEUTROPHIL\_DN |
| Enrichment Score (ES) | 0.4693009 |
| Normalized Enrichment Score (NES) | 1.926048 |
| Nominal p-value | 0.0034542314 |
| FDR q-value | 0.65467465 |
| FWER p-Value | 1.0 |
Table: GSEA Results Summary

  

Fig 1: Enrichment plot: GSE27786\_ERYTHROBLAST\_VS\_NEUTROPHIL\_DN      
 Profile of the Running ES Score & Positions of GeneSet Members on the Rank Ordered List

  

| SYMBOL | TITLE | RANK IN GENE LIST | RANK METRIC SCORE | RUNNING ES | CORE ENRICHMENT || 1 | 51111 | KMT5B | 2 | 1.217 | 0.1091 | Yes |
| 2 | 10513 | APPBP2 | 28 | 1.000 | 0.1821 | Yes |
| 3 | 57594 | HOMEZ | 44 | 0.933 | 0.2561 | Yes |
| 4 | 23054 | NCOA6 | 77 | 0.838 | 0.3094 | Yes |
| 5 | 403313 | PLPP6 | 274 | 0.615 | 0.2246 | Yes |
| 6 | 8125 | ANP32A | 326 | 0.578 | 0.2405 | Yes |
| 7 | 4297 | KMT2A | 337 | 0.573 | 0.2854 | Yes |
| 8 | 51535 | PPHLN1 | 361 | 0.560 | 0.3198 | Yes |
| 9 | 57109 | REXO4 | 370 | 0.552 | 0.3643 | Yes |
| 10 | 9815 | GIT2 | 394 | 0.540 | 0.3969 | Yes |
| 11 | 5814 | PURB | 399 | 0.536 | 0.4427 | Yes |
| 12 | 53944 | CSNK1G1 | 485 | 0.484 | 0.4256 | Yes |
| 13 | 9830 | TRIM14 | 486 | 0.480 | 0.4693 | Yes |
| 14 | 6197 | RPS6KA3 | 643 | 0.403 | 0.3940 | No |
| 15 | 1717 | DHCR7 | 836 | 0.288 | 0.2823 | No |
| 16 | 2037 | EPB41L2 | 943 | -0.365 | 0.2394 | No |
| 17 | 79094 | CHAC1 | 949 | -0.367 | 0.2692 | No |
| 18 | 91947 | ARRDC4 | 1282 | -0.671 | 0.0919 | No |
Table: GSEA details [plain text format]

  

Fig 2: GSE27786\_ERYTHROBLAST\_VS\_NEUTROPHIL\_DN      
 Blue-Pink O' Gram in the Space of the Analyzed GeneSet

  

Fig 3: GSE27786\_ERYTHROBLAST\_VS\_NEUTROPHIL\_DN: Random ES distribution      
 Gene set null distribution of ES for **GSE27786\_ERYTHROBLAST\_VS\_NEUTROPHIL\_DN**
